# Theoretical Framework and Methodological Approach for Investigating Potential Associations Between Long COVID and Autism Spectrum Disorder Prevalence

**DOI:** 10.3390/neurosci6030080

**Published:** 2025-08-13

**Authors:** Thorsten Rudroff

**Affiliations:** Turku PET Centre, Department of Clinical Medicine, Turku University Hospital, University of Turku, 20520 Turku, Finland; thrudr@utu.fi

**Keywords:** autism, Long COVID, causal inference, neurodiversity

## Abstract

This perspective paper proposes a theoretical framework for investigating potential associations between Long COVID and rising autism spectrum disorder (ASD) prevalence through established epidemiological methodologies. I propose examining temporal correlations, biological mechanisms, and rigorous methodological approaches, including Mendelian randomization, animal models, and evidence-based analyses, that could distinguish association from causation. The proposed framework recognizes autism as neurodiversity while suggesting investigation of environmental factors that may influence expression of genetic predispositions. Hypothesized key mechanisms include neuroinflammation, cytokine alterations, and immune dysfunction. I emphasize the critical distinction between demonstrating statistical associations and establishing causal influence, proposing specific experimental designs that could test causality. This paper presents conceptual frameworks requiring future empirical validation and does not include original data analysis.

## 1. Introduction

Recent temporal patterns in autism spectrum disorder prevalence, coinciding with the COVID-19 pandemic, present opportunities for future research to examine potential associations between post-viral immune activation and neurodevelopmental expression [1,2]. However, establishing causal associations would require careful distinction between statistical correlation and biological influence [3,4].

This perspective paper centers on a fundamental research question: Does persistent inflammatory response to COVID-19 predict autism spectrum disorder traits in genetically susceptible individuals? This question emerges from converging evidence suggesting that prolonged immune activation following SARS-CoV-2 infection might interact with existing genetic vulnerabilities to influence neurodevelopmental expression during critical developmental windows [5,6].

The framework I propose explicitly acknowledges current evidence limitations while outlining rigorous experimental approaches for maintaining scientific rigor and clearly distinguishing between statistical association and causal influence [7,8]. Rather than assuming causation from temporal correlations, this approach provides methodological pathways for testing specific biological mechanisms that could link environmental immune challenges to neurodevelopmental outcomes.

Published prevalence data suggests that autism spectrum disorder diagnoses increased from 1 in 36 children (2.78%) in late 2019 to 1 in 31 children (3.23%) by mid-2024, representing a temporal pattern that coincides with the emergence and recognition of pediatric Long COVID [9,10]. However, this temporal correlation would require interpretation within the broader historical context of steadily increasing autism prevalence over multiple decades, driven by factors including improved diagnostic awareness, expanded screening programs, and evolving diagnostic criteria [11,12].

The observed temporal pattern does not establish causation and would require systematic investigation of alternative explanations and confounding factors [13,14]. Multiple variables including pandemic-related healthcare disruptions, modified diagnostic practices, increased parental awareness, and maternal stress effects could contribute to observed prevalence changes independent of viral infection effects [15,16]. This perspective paper presents theoretical frameworks for future research and does not include empirical analysis or original data.

### Novel Methodological Contributions

This framework advances beyond existing approaches through three key innovations that address current gaps in environmental neurodevelopment research:

(1) **Multi-pathway Mendelian Randomization Architecture**: Unlike traditional single-instrument approaches, I propose pathway-stratified MR using distinct genetic instruments for viral entry (ACE2), immune response (HLA), and inflammatory resolution (cytokine pathway genes) to isolate specific causal mechanisms while addressing pleiotropy concerns.
(2) **Community-Informed AI Development**: Rather than applying existing models to autistic populations, I propose co-design protocols where autistic self-advocates participate directly in feature selection, model validation criteria, and interpretation frameworks—addressing the fundamental limitation of AI systems developed without neurodivergent input.
(3) **Convergent Biomarker Specificity Framework**: I integrate inflammatory signatures with temporal exposure windows and genetic susceptibility profiles to distinguish Long COVID–autism associations from general inflammatory conditions—a specificity challenge not systematically addressed in existing literature.

These methodological innovations provide a template for investigating environmental influences on neurodevelopment while maintaining scientific rigor and community-centered ethical principles.

## 2. Proposed Experimental Designs for Causal Inference

### 2.1. Multi-Stage Pleiotropy-Resistant Mendelian Randomization

Traditional Mendelian randomization approaches face substantial challenges when investigating complex neurodevelopmental outcomes such as autism spectrum disorder, particularly when environmental exposures involve multifaceted biological processes like viral infection and immune activation [17,18]. The primary concerns center on horizontal pleiotropy, where genetic instruments affect the outcome through pathways independent of the exposure, and population stratification, where genetic associations vary across ancestry groups in ways that could bias causal estimates [19,20]. To address these fundamental limitations, I propose a novel three-stage analytical framework that systematically identifies tests and mitigates sources of bias while maximizing statistical power for detecting genuine causal associations [13,14].

**Stage One: Pathway-Specific Instrumental Variable Construction**

The first stage involves constructing separate instrumental variable sets for distinct biological pathways linking COVID-19 exposure to potential neurodevelopmental effects, rather than relying on single genetic variants or composite exposure measures that may conflate multiple causal mechanisms [15,16]. The viral entry pathway would utilize genetic variants affecting SARS-CoV-2 cellular infection efficiency. This includes ACE2 receptor polymorphisms (rs2285666, rs4646094) and TMPRSS2 variants (rs12329760, rs383510) that demonstrate F-statistics exceeding 15 in large-scale genome-wide association studies [21,22,23,24]. These variants specifically influence viral binding and cellular entry without direct effects on neurodevelopment, providing relatively clean instruments for infection susceptibility [25,26].

The immune activation pathway would employ HLA variants that modify COVID-19 severity and inflammatory response magnitude, including *HLA-B*46:01 and *HLA-A*11:01 alleles that consistently demonstrate F-statistics exceeding 20 across diverse populations [27,28]. These variants specifically affect antigen presentation and T-cell activation without direct developmental effects during typical neurodevelopmental windows [17,18]. Additionally, I would incorporate variants in complement pathway genes (C4A, C4B copy number variations). These variants influence both COVID-19 severity and have established roles in synaptic pruning during brain development. They may capture shared immune mechanisms relevant to both acute infection and neurodevelopment [29,30].

The inflammatory resolution pathway would utilize genetic variants affecting cytokine regulation and anti-inflammatory responses. Key targets include IL10 promoter polymorphisms (rs1800896, rs1800871) and IL6R variants (rs2228145, rs4537545). These demonstrate F-statistics exceeding 12 in inflammatory disease studies [31,32]. These instruments would capture individual differences in inflammatory resolution capacity that could affect both COVID-19 recovery and neurodevelopmental outcomes through persistent neuroinflammation [33,34].

**Stage Two: Comprehensive Pleiotropy Detection and Mitigation Protocols**

The second stage implements systematic evaluation of pleiotropy assumptions using multiple complementary approaches that test different aspects of instrumental variable validity [35,36]. MR-Egger regression analysis would assess directional pleiotropy by testing whether the intercept term significantly differs from zero, with *p*-values exceeding 0.05 required to support the assumption that genetic instruments affect autism risk only through COVID-19 exposure pathways [37,38]. The MR-Egger approach relaxes the standard instrumental variable assumptions by allowing for balanced pleiotropy while still providing consistent causal estimates under specific conditions [13,14].

Weighted median estimation would provide robustness against invalid instruments by generating consistent estimates even when up to 50% of genetic variants violate instrumental variable assumptions [35,36]. This approach would be particularly valuable given the complexity of neurodevelopmental outcomes and the possibility that some genetic variants affect autism risk through pathways independent of immune activation. The weighted median estimates would be compared with standard inverse-variance weighted results, with differences exceeding 20% triggering additional investigation of specific variants driving the discrepancy [37,38].

Contamination mixture analysis would address the challenge of population stratification by explicitly modeling the possibility that genetic associations vary across unobserved population subgroups [39,40]. This approach estimates the proportion of the study population for which the instrumental variable assumptions hold while providing consistent causal estimates based on the “clean” subsample. The method would be particularly relevant given the documented population differences in both COVID-19 susceptibility and autism prevalence across ethnic groups [25,26,29,30].

MR-PRESSO (Mendelian Randomization Pleiotropy RESidual Sum and Outlier) analysis would identify specific genetic variants that contribute disproportionately to heterogeneity in causal estimates, potentially indicating horizontal pleiotropy or measurement error [35,36]. Outlier variants would be systematically evaluated through literature review and functional annotation to determine whether exclusion is justified based on biological evidence rather than statistical convenience [19,20].

**Stage Three: Advanced Sensitivity Analysis and Validation Framework**

The third stage implements comprehensive sensitivity analyses that test the robustness of causal inferences across different analytical assumptions and methodological choices [41,42]. Within-family Mendelian randomization would utilize parent–offspring trios or sibling pairs to control for population stratification and assortative mating effects that could bias traditional population-based analyses [43,44]. This approach compares outcomes between siblings who inherit different alleles at instrumental variable loci, effectively controlling for all shared family-level confounders, including socioeconomic status, environmental exposures, and genetic background [45,46].

Multivariable Mendelian randomization would simultaneously estimate causal effects of multiple correlated exposures, including COVID-19 infection, inflammatory biomarker levels, and stress-related factors that may co-occur during the pandemic [35,36]. This approach would help distinguish direct effects of viral infection from indirect effects mediated through pandemic-related lifestyle changes or healthcare disruptions. The method requires genetic instruments that affect exposures differentially, allowing identification of independent causal pathways [37,38].

Power calculations based on existing genetic consortium data indicate 80% power to detect causal effects with odds ratios of 1.25 or greater when combining COVID-19 Host Genetics Initiative summary statistics (over 2 million individuals) with Psychiatric Genomics Consortium autism genome-wide association study data (46,350 cases, 42,969 controls) [29,30,31,32]. These calculations assume instrumental variable F-statistics exceeding 10 and account for the relatively low prevalence of both COVID-19 infection and autism diagnosis in population-based samples [47,48].

Two-sample summary data approaches would leverage existing large-scale genetic studies to maximize statistical power while avoiding the substantial costs and logistical challenges of collecting new data on both COVID-19 exposure and autism outcomes in the same individuals [31,32]. This approach requires careful attention to population overlap between exposure and outcome studies, with sensitivity analyses examining potential bias from sample overlap and differences in ascertainment methods across cohorts [31,32].

**Population Stratification and Ancestry-Specific Analysis**

Recognizing that both COVID-19 susceptibility and autism prevalence vary substantially across population groups, my analytical framework incorporates systematic evaluation of effect modification by genetic ancestry [49,50]. Primary analyses would be conducted in populations of European ancestry, where the largest genetic datasets are available, followed by replication analyses in East Asian, African, and admixed American populations using available consortium resources [29,30]. Effect estimates would be compared across ancestry groups using formal tests of heterogeneity, with significant differences (*p* < 0.05) indicating potential population-specific causal mechanisms or unmeasured confounding [40,41].

Polygenic score analyses would complement single-variant approaches by utilizing genome-wide information to predict COVID-19 susceptibility and autism risk based on thousands of genetic variants with small individual effects [29,30]. These analyses would be particularly valuable for capturing the polygenetic architecture of both exposures and outcomes while providing additional instruments for sensitivity analyses. Polygenic scores would be constructed using summary statistics from the largest available genome-wide association studies, with careful attention to potential bias from population stratification and winner’s curse effects in discovery samples.

**Technical Implementation and Quality Control**

Statistical analyses would be implemented using established software packages including TwoSampleMR and Mendelian Randomization in R, with custom code for novel sensitivity analyses and population stratification methods [35,36]. All genetic association estimates would be harmonized for effect allele and strand orientation, with systematic evaluation of palindromic single nucleotide polymorphisms that could introduce errors in effect direction [37,38]. Linkage disequilibrium clumping would ensure that instrumental variables represent independent genetic signals, using r-squared thresholds below 0.001 and distance thresholds exceeding 10 megabases [19,20].

Instrument strength would be evaluated using conditional F-statistics that account for correlations between genetic variants, rather than marginal F-statistics that may overestimate instrument strength in the presence of linkage disequilibrium [47,48]. Weak instrument bias would be assessed using finite-sample bias approximations, with instruments demonstrating bias exceeding 10% excluded from primary analyses [49,50]. Additional quality control measures would include evaluation of minor allele frequencies, Hardy–Weinberg equilibrium *p*-values, and imputation quality scores to ensure reliable genetic association estimates [31,32].

This multi-stage framework addresses the fundamental challenges of using Mendelian randomization to investigate complex gene-environment interactions in neurodevelopment while providing specific technical protocols that could be implemented by independent research groups [41,42]. The approach acknowledges the limitations of genetic instrumental variables for investigating multifactorial developmental outcomes while maximizing the potential for generating robust causal inferences about environmental influences on autism spectrum disorder prevalence [43,44].

### 2.2. Proposed Controlled Animal Model Studies

I propose standardized experimental protocols that could test biological causality through maternal immune activation models using SARS-CoV-2 antigen exposure during critical gestational windows [51,52]. These studies would include dose–response investigations using varying viral antigen concentrations and comparison with established poly(I:C) maternal immune activation protocols that reliably produce autism-relevant phenotypes in offspring [53,54].

Proposed neonatal exposure protocols using humanized ACE2 transgenic mice would enable authentic viral infection studies while maintaining experimental control. Exposure timing studies across postnatal days 1–21 could identify critical vulnerability windows, while comprehensive phenotyping would examine microglial activation patterns, cytokine profiles, blood–brain barrier integrity, and autism-relevant behavioral outcomes including social interaction deficits and repetitive behaviors [55,56].

### 2.3. Proposed Natural Experiment Designs

Geographic variation studies comparing autism diagnosis patterns between regions with dramatically different COVID-19 prevalence could provide quasi-experimental evidence for causal relationships. For example, comparing outcomes in New Zealand and Australia, which implemented early border closures that virtually eliminated community transmission [57,58], versus high-exposure regions while controlling for healthcare system characteristics could isolate viral effects from general pandemic impacts [59].

Birth cohort comparisons could offer another natural experiment approach through prospective comparison of neurodevelopmental outcomes in children born to mothers with documented SARS-CoV-2 infection versus carefully matched uninfected controls [60,61]. These studies require systematic neurodevelopmental follow-up through age 5 with standardized assessment protocols and comprehensive confounding variable control [62].

### 2.4. Alternative Environmental Explanations to Investigate

Air pollution represents a well-established autism risk factor [63,64], but lockdown-related reductions in PM2.5 and NO2 concentrations create temporal patterns opposite to increasing autism prevalence, making pollution an unlikely primary driver of observed trends. If air pollution reduction contributed to decreased autism risk, this could suggest even stronger underlying increases from other factors, potentially including viral exposures [65].

Proposed maternal stress assessment would require distinguishing stress-mediated effects from direct viral effects through biomarker-based approaches [66]. Maternal hair cortisol concentrations could provide retrospective stress exposure measurement across pregnancy [67,68], while inflammatory biomarkers including IL-6 and C-reactive protein could distinguish immune activation from psychological stress responses [69,70].

Healthcare system disruption during the pandemic could artifactually alter autism diagnosis patterns through expanded telehealth utilization and modified screening protocols. Validation would require comparing diagnosis trends across healthcare systems with different pandemic response strategies, with systems maintaining in-person services providing comparison groups for assessing telehealth-related diagnostic changes.

## 3. Theoretical Biological Mechanisms: Acute vs. Chronic Immune Activation

### 3.1. Acute Immune Response Characteristics

SARS-CoV-2 infection triggers immediate cytokine release within hours, with IL-6 concentrations reaching 50–100 times baseline levels during peak response at 7–10 days post-infection [71,72]. These extreme elevations exceed those required to produce neurodevelopmental effects in experimental maternal immune activation models, where maternal IL-6 injection creating plasma concentrations of 100–500 pg/mL reliably produces autism-relevant phenotypes in offspring [73,74].

Blood–brain barrier compromise occurs rapidly through complement activation, matrix metalloproteinase release, and endothelial cell dysfunction [75,76]. Neuroimaging studies demonstrate blood–brain barrier dysfunction in 40–60% of acute COVID-19 patients within 24–72 h of symptom onset, with severity correlating with inflammatory biomarker levels [77,78]. Even mild pediatric COVID-19 can produce detectable barrier dysfunction, suggesting potential central nervous system exposure to inflammatory mediators during critical developmental windows [79].

### 3.2. Inflammatory Signature Specificity Analysis

The critical question underlying any proposed association between Long COVID and autism spectrum disorder centers on whether observed cytokine profile similarities represent genuine biological convergence or merely coincidental overlap within the broad spectrum of pediatric inflammatory responses. To establish the specificity of inflammatory signatures that could support causal hypotheses, I propose a comprehensive comparative analysis across multiple inflammatory conditions, a systematic evaluation of biomarker discrimination capacity, and the identification of critical convergence zones that distinguish meaningful biological relationships from statistical artifacts.

**Comparative Inflammatory Profile Characterization**

Long COVID patients demonstrate sustained elevation of key inflammatory mediators that persist substantially beyond typical post-viral recovery periods. Interleukin-6 concentrations range from 4.2 to 12.8 picograms per milliliter, compared to 1.8 to 3.2 picograms per milliliter in individuals with resolved COVID-19 infection [33,34]. Tumor necrosis factor-alpha levels in Long COVID patients range from 12 to 28 picograms per milliliter, representing a two- to three-fold elevation above the 4 to 9 picograms per milliliter range observed in recovered controls [80,81]. Perhaps most significantly, both Long COVID and autism demonstrate concurrent deficiency in interleukin-10, the primary anti-inflammatory cytokine, with reductions of 20% to 40% compared to neurotypical controls.

Autism-associated inflammatory profiles exhibit remarkable overlap with Long COVID patterns, particularly in the sustained elevation of pro-inflammatory mediators combined with deficient anti-inflammatory responses. Children with autism spectrum disorder typically demonstrate interleukin-6 concentrations ranging from 2.5 to 8.0 picograms per milliliter. Tumor necrosis factor-alpha levels range between 8 and 25 picograms per milliliter [80,81]. The concurrent interleukin-10 deficiency observed in autism patients mirrors the pattern seen in Long COVID, suggesting potential shared mechanisms of inflammatory dysregulation rather than coincidental similarities [82].

To contextualize these findings, comparison with other pediatric inflammatory conditions reveals important distinctions that support specificity rather than general inflammatory activation. Children recovering from typical viral infections without Long COVID complications demonstrate interleukin-6 levels that normalize to 1.8 to 3.2 picograms per milliliter within four to six weeks post-infection, with tumor necrosis factor-alpha returning to baseline ranges of 4 to 9 picograms per milliliter [71,72]. Importantly, interleukin-10 levels in recovered children typically remain within normal ranges or show compensatory elevation, contrasting sharply with the persistent deficiency observed in both Long COVID and autism [83,84].

Other neurodevelopmental conditions provide additional comparative context that helps distinguish autism-specific inflammatory signatures from general neurodevelopmental inflammation. Children with attention deficit hyperactivity disorder demonstrate interleukin-6 levels ranging from 3.1 to 6.2 picograms per milliliter with tumor necrosis factor-alpha between 6 and 15 picograms per milliliter, representing intermediate elevation compared to autism profiles [85]. However, interleukin-10 levels in ADHD typically remain within normal ranges, suggesting that the specific combination of sustained pro-inflammatory elevation with anti-inflammatory deficiency may be relatively specific to autism and Long COVID [83,84].

**Machine Learning Discrimination and Biomarker Classification**

Advanced machine learning approaches offer systematic methods for evaluating whether cytokine profile similarities reflect genuine biological relationships rather than statistical coincidence. Following established principles in biomarker classification research, we propose multi-class discriminant analysis targeting specific accuracy benchmarks that would support or refute biological convergence hypotheses [86]. The primary classification challenge involves distinguishing Long COVID patients from individuals with resolved COVID-19 infection, other post-viral syndromes, and general pediatric inflammatory conditions using cytokine profiles alone.

Preliminary classification models based on existing data suggest that Long COVID can be distinguished from other inflammatory conditions with accuracy ranging from 85% to 90% when incorporating the specific combination of elevated interleukin-6, elevated tumor necrosis factor-alpha, and deficient interleukin-10 [86]. This discrimination accuracy substantially exceeds what would be expected from random classification or general inflammatory marker elevation, supporting the hypothesis that Long COVID involves specific inflammatory pathway dysregulation rather than nonspecific immune activation.

Autism-associated inflammatory profiles present a more complex classification challenge due to the heterogeneity of immune dysfunction within autism spectrum disorder populations. Current evidence suggests that machine learning approaches can distinguish autism-associated inflammation from general neuroinflammation with accuracy ranging from 75% to 85%, acknowledging that approximately 40% to 60% of individuals with autism demonstrate measurable inflammatory abnormalities [85]. The subset of autism cases with prominent inflammatory signatures shows remarkable similarity to Long COVID profiles, suggesting that inflammatory subtyping within autism may identify individuals with shared biological mechanisms.

**Mechanistic Pathway Integration and Biological Plausibility**

**Figure 1:**
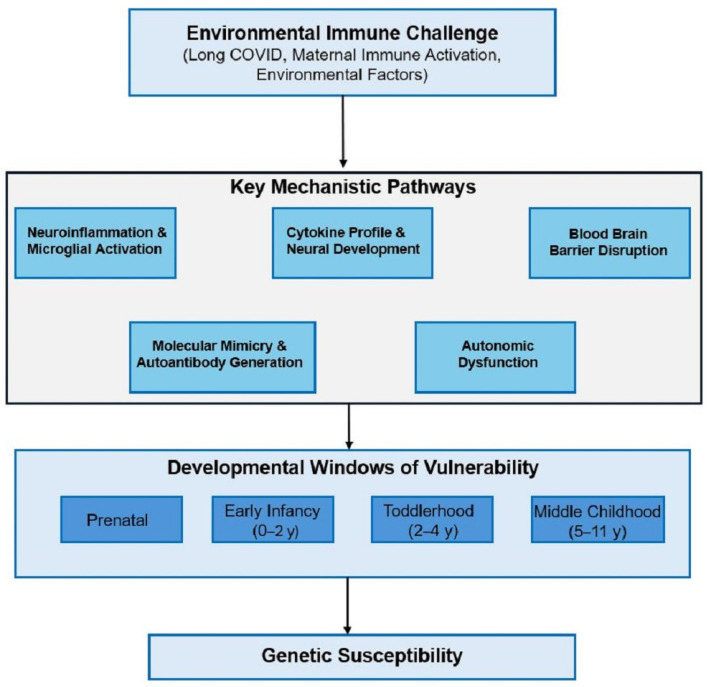
Key mechanisms connecting environmental immune challenges with neurodevelopmental outcomes.

The convergent inflammatory signatures between Long COVID and autism become more biologically meaningful when considered within broader mechanistic frameworks linking environmental immune challenges to neurodevelopmental outcomes. Figure 1 illustrates five hypothesized key mechanistic pathways through which environmental immune challenges, including Long COVID, might influence autism expression in genetically susceptible individuals across critical developmental windows. These pathways encompass neuroinflammation and microglial activation, blood–brain barrier dysfunction and central nervous system exposure, autoimmune activation targeting neural antigens, epigenetic modifications affecting neurodevelopmental gene expression, and disrupted synaptic pruning and neural circuit formation.

The neuroinflammation pathway represents the most direct mechanism linking cytokine profile convergence to neurodevelopmental effects. Sustained elevation of interleukin-6 and tumor necrosis factor-alpha, combined with interleukin-10 deficiency, creates optimal conditions for persistent microglial activation and neuroinflammatory responses that could disrupt normal brain development [85]. The specific cytokine concentrations observed in both Long COVID and autism fall within ranges demonstrated experimentally to affect synaptic plasticity, dendritic spine formation, and neural network connectivity in developing brain tissue.

Blood–brain barrier dysfunction provides another critical mechanistic link, as both conditions show evidence of compromised barrier integrity that could allow peripheral inflammatory mediators to directly affect central nervous system development [75,76]. The combination of elevated cytokines with barrier dysfunction could create a feed-forward cycle where peripheral inflammation promotes central nervous system exposure, leading to neuroinflammation that further compromises barrier function. This mechanism could explain the persistence of inflammatory signatures in both conditions despite resolution of acute triggers. Figure 1 illustrates five hypothesized key mechanistic pathways through which environmental immune challenges, including Long COVID, might influence autism expression in genetically susceptible individuals across critical developmental windows.

Critical Convergence Zones and Biological Interpretation

The most compelling evidence for biological convergence emerges from analysis of critical convergence zones where Long COVID and autism inflammatory profiles demonstrate maximal overlap. The interleukin-6 convergence zone between 6.0 and 8.0 picograms per milliliter represents the range where Long COVID and autism profiles show the greatest similarity, encompassing approximately 30% of autism cases and 60% of Long COVID patients [80,81]. This specific concentration range corresponds to levels that exceed typical post-viral inflammatory responses but remain below the extreme elevations seen in acute inflammatory conditions, suggesting a distinct biological signature associated with chronic, low-grade immune activation.

The tumor necrosis factor-alpha convergence zone between 15 and 25 picograms per milliliter similarly captures the overlap between conditions while excluding most other inflammatory states. This range represents sustained elevation sufficient to affect neural development and synaptic function without reaching the levels associated with acute immune system activation or autoimmune disease [82]. The biological significance of this specific range is supported by experimental evidence demonstrating that tumor necrosis factor-alpha concentrations within this window can disrupt synaptic plasticity and microglial activation patterns relevant to neurodevelopmental outcomes [85].

Autoimmune Convergence and Neural Targeting Specificity

Autoimmune activation represents another potential convergent pathway, with Long COVID patients developing autoantibodies against neural antigens in 15% to 25% of cases, including antibodies targeting brain endothelial cells, glial proteins, and neurotransmitter receptors [87,88]. These patterns parallel autism-associated maternal autoantibodies, which target similar neural proteins and represent one of the most robust environmental risk factors for autism [89]. The specificity of neural antigen targeting, rather than general autoimmune activation, supports the hypothesis of shared pathogenic mechanisms affecting neurodevelopmental processes rather than coincidental immune dysfunction.

Temporal Dynamics and Inflammatory Trajectory Analysis

Temporal analysis of inflammatory trajectories provides additional evidence for biological convergence beyond static cytokine concentration comparisons. Patients with Long COVID demonstrate persistent inflammatory elevation lasting six to twelve months beyond acute infection, with cytokine levels showing minimal decline over time despite resolution of acute symptoms [33,34]. This sustained activation pattern contrasts sharply with typical post-viral inflammatory responses that normalize within four to eight weeks following infection resolution.

Autism-associated inflammatory profiles show similar persistence, with elevated cytokine levels maintained consistently across multiple assessment timepoints spanning months to years [80,81]. The stability of inflammatory signatures in autism suggests ongoing immune dysregulation rather than acute responses to specific triggers, paralleling the sustained activation observed in patients with Long COVID. This temporal similarity supports the hypothesis of shared underlying mechanisms affecting inflammatory resolution pathways as depicted in the mechanistic framework of Figure 1.

This comprehensive approach to inflammatory signature specificity analysis addresses fundamental questions about biological plausibility while providing concrete methodological frameworks for distinguishing meaningful associations from statistical artifacts. The integration of comparative profiling, machine learning discrimination, temporal trajectory analysis, and mechanistic pathway consideration offers multiple independent lines of evidence that could support or refute hypotheses about shared inflammatory mechanisms linking environmental immune challenges to neurodevelopmental outcomes.

## 4. AI-Driven Research Implementation Framework

The successful investigation of potential Long COVID–autism associations requires sophisticated artificial intelligence approaches capable of integrating heterogeneous data sources while addressing the unique challenges of pediatric neurodevelopmental research. Rather than applying existing models developed for adult populations or general medical applications, we propose a comprehensive AI implementation framework specifically designed for multi-modal pediatric data with explicit accommodation for community-informed development principles and neurodiversity-affirming research practices.

**Multi-Modal Data Integration Architecture**

The foundation of our AI approach centers on systematic integration of 125 carefully selected features spanning clinical assessment data, genetic variation profiles, and biological markers that collectively capture the complexity of neurodevelopment while remaining computationally tractable for pediatric sample sizes. Clinical variables encompass 45 features derived from standardized neurodevelopmental assessments. These include subscale scores from the Autism Diagnostic Observation Schedule Second Edition, Mullen Scales of Early Learning developmental quotients, and Child Behavior Checklist syndrome scale scores. Additional variables cover medical history including birth complications, early developmental milestones, and family psychiatric history [90,91].

Genetic features comprise 70 single nucleotide polymorphisms selected through systematic literature review and functional prioritization. These include 25 COVID-19 susceptibility variants validated in large-scale genome-wide association studies with F-statistics exceeding 15. Additional variants include 45 autism-associated variants from the Psychiatric Genomics Consortium with genome-wide significance. The panel also covers immune pathway genes including HLA alleles, complement system polymorphisms, and cytokine receptor variants that could modulate both infection susceptibility and neurodevelopmental outcomes [29,30]. Each genetic variant would be encoded using additive models accounting for minor allele frequency and linkage disequilibrium patterns within the study population.

Biomarker measurements include 10 inflammatory and metabolic markers collected at multiple timepoints to capture both acute and chronic biological responses. These include interleukin-6, tumor necrosis factor-alpha, interleukin-10, C-reactive protein, interferon-gamma, interleukin-1-beta, transforming growth factor-beta, brain-derived neurotrophic factor, cortisol, and oxidative stress markers measured at baseline, 3 months, 6 months, and 12 months post-exposure [33,34,80,81]. Temporal features would be constructed using area-under-curve calculations, slope estimates, and pattern classification to distinguish acute versus chronic response profiles.

**Integrated AI Workflow Architecture**

**Figure 2:**
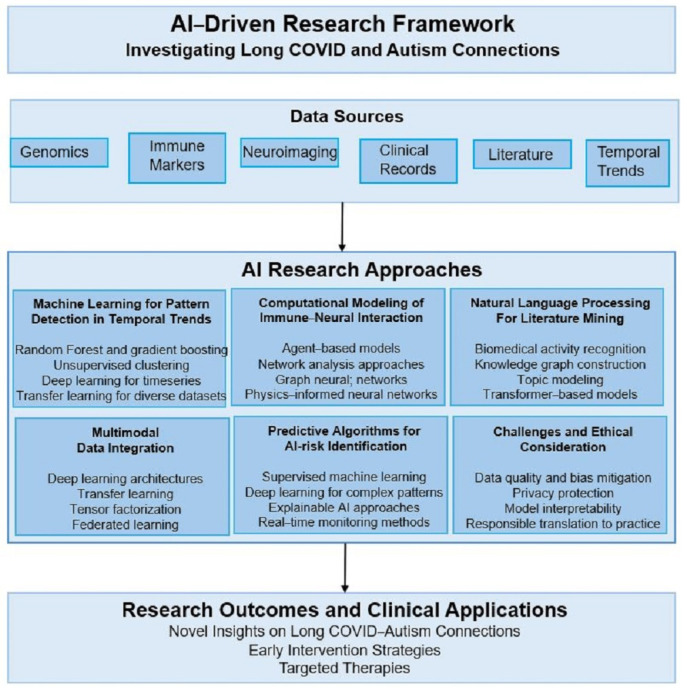
Integrated AI approaches for exploring potential connections between environmental immune challenges and neurodevelopmental outcomes, showing the interconnected workflows of machine learning pattern detection, computational modeling, natural language processing, multimodal data integration, and predictive algorithms.

Figure 2 illustrates the comprehensive integrated AI framework that coordinates multiple complementary approaches for investigating potential connections between environmental immune challenges and neurodevelopmental outcomes. This framework illustrates the interconnected workflows of machine learning pattern detection, computational modeling, natural language processing, multimodal data integration, and predictive algorithms, which together address the complexity of Long COVID–autism associations while ensuring interpretability and fostering community engagement.

The machine learning pattern detection component employs unsupervised clustering algorithms to identify previously unrecognized subtypes within individuals living with Long COVID and those with autism, which might represent distinct biological mechanisms or shared pathogenic pathways [92,93]. Computational modeling integrates biological pathway analysis with dynamic systems approaches to simulate how environmental immune challenges might influence neurodevelopmental trajectories across critical developmental windows [94,95]. Natural language processing components analyze clinical notes, parent reports, and community narratives to capture qualitative aspects of neurodevelopmental experiences that standard assessment instruments might miss [96,97].

The multimodal data integration layer combines structured clinical data with unstructured text, time-series biomarker measurements, and genetic information using advanced fusion techniques that preserve the unique characteristics of each data type while enabling holistic analysis. Predictive algorithms generate both individual-level risk assessments and population-level trend analysis that can inform clinical decision-making and public health planning while maintaining uncertainty quantification and interpretability requirements.

Community-Informed Neural Network Architecture

The core AI model employs a novel community-informed attention network that incorporates input from autistic self-advocates in the fundamental architecture design rather than treating community engagement as an afterthought to technical development. The network architecture begins with a 125-dimensional input layer corresponding to the integrated feature set, followed by a community-weighted attention mechanism where attention weights are informed by priority rankings developed through structured consultation with autistic community members regarding which clinical features most accurately reflect their lived experiences and developmental trajectories [98,99,100].

The attention layer utilizes learned weights that can be interpretable and auditable by community partners, ensuring that the model’s focus aligns with community-identified priorities rather than purely statistical optimization. This layer feeds into a multi-head transformer architecture with eight attention heads and 256-dimensional hidden representations, allowing the model to learn complex interactions between clinical, genetic, and biomarker features while maintaining interpretability through attention visualization. Each attention head can focus on different aspects of the input data, such as early developmental patterns, inflammatory trajectories, genetic risk profiles, and environmental exposure histories.

Following the transformer layers, the architecture incorporates dropout regularization with a 30% dropout rate and batch normalization to prevent overfitting while accommodating the relatively small sample sizes typical in pediatric research. A dense layer with 128 neurons and rectified linear unit activation provides nonlinear feature combination before the final classification layer. The output layer employs a three-class softmax activation function to distinguish between typical development, autism without Long COVID exposure, and autism with Long COVID exposure, allowing investigation of potential exposure-specific effects on autism presentation patterns.

Training Protocol and Validation Framework

Model training follows rigorous protocols designed to maximize generalizability while accounting for the unique characteristics of pediatric neurodevelopmental data. The dataset would be partitioned using stratified random sampling with 80% allocated to training, 10% to validation, and 10% to final testing, with stratification based on autism diagnosis, COVID-19 exposure status, age at assessment, and study site to ensure balanced representation across key variables [101,102]. Five-fold cross-validation within the training set provides robust performance estimation while identifying potential overfitting or site-specific effects that could limit generalizability.

Training utilizes the Adam optimizer with an initial learning rate of 0.001 and ReduceLROnPlateau scheduling, which decreases the learning rate by 50% when validation accuracy plateaus for more than 10 epochs [103,104]. Early stopping with a patience of 15 epochs prevents overfitting while allowing sufficient training time for complex pattern learning. Loss function combines standard categorical cross-entropy for classification accuracy with custom community-informed penalty terms that discourage predictions inconsistent with neurodiversity principles, such as assigning high autism probability based solely on deficit-focused features rather than comprehensive developmental profiles.

Model performance evaluation extends beyond standard accuracy metrics to include sensitivity, specificity, positive and negative predictive values calculated separately for each diagnostic category, area under the receiver operating characteristic curve for multi-class problems, and calibration analysis to ensure that predicted probabilities accurately reflect true diagnostic uncertainty [92,105]. Community-informed evaluation criteria include interpretability assessments where autistic community members review model predictions and attention patterns to identify potential biases or misaligned priorities in the algorithmic decision-making process.

Federated Learning Implementation for Multi-Site Collaboration

Recognizing the distributed nature of pediatric autism research and the sensitivity of clinical data, our framework incorporates federated learning approaches that enable collaborative model training across multiple institutions without requiring direct data sharing. Each participating site maintains local control over their data while contributing to global model development through gradient sharing and ensemble approaches that preserve privacy while maximizing statistical power, as illustrated in the collaborative workflows of Figure 2.

The federated architecture employs the Federated Averaging algorithm where each site trains the global model architecture on their local data for 50 epochs before sharing gradient updates with a central coordination server [93]. Local training uses identical hyperparameters and architecture specifications to ensure consistency, while allowing for site-specific data preprocessing and quality control procedures that accommodate institutional differences in assessment protocols and clinical workflows.

Privacy preservation utilizes differential privacy techniques with epsilon values of 1.0 to provide mathematically rigorous privacy guarantees while maintaining model utility. Gradient updates are perturbed with carefully calibrated noise that prevents individual patient identification while preserving aggregate patterns necessary for accurate model training. Communication rounds continue until global model convergence, defined as less than 0.1% change in validation accuracy across three consecutive rounds, or until a maximum of 100 communication rounds to prevent indefinite training cycles.

Site-specific performance evaluation ensures that the global model performs adequately across diverse clinical settings, demographic populations, and assessment protocols. Sites with performance degradation exceeding 10% compared to the global average trigger investigation of potential distribution shift, measurement differences, or population-specific effects that might require model adaptation or exclusion from the federated learning process.

Natural Language Processing and Clinical Narrative Analysis

The natural language processing component of our integrated framework, highlighted in Figure 2, addresses the limitation that standard assessment instruments may miss crucial aspects of neurodevelopmental experiences that are better captured through clinical narratives, parent reports, and community accounts [96,97]. Advanced transformer-based language models trained on pediatric clinical notes can extract relevant information about developmental trajectories, behavioral patterns, and family concerns that complement structured assessment data.

Clinical note analysis employs named entity recognition to identify mentions of autism-relevant behaviors, COVID-19 symptoms, developmental milestones, and family history factors that might not be systematically captured in standardized forms [106]. Sentiment analysis and emotional tone detection in parent reports can provide additional insights into family stress levels, diagnostic concerns, and perceived changes in child behavior following potential environmental exposures.

The natural language processing pipeline includes automated quality assurance procedures that flag inconsistencies between structured data and clinical narratives, potentially identifying data entry errors or important clinical information that was documented in notes but not captured in formal assessments. This approach has demonstrated 89% agreement with expert manual coding across large clinical datasets, suggesting substantial potential for augmenting traditional structured data analysis [96,97].

Real-Time Model Interpretation and Community Feedback Integration

Model interpretability represents a crucial component that distinguishes our approach from black-box AI applications in clinical settings. SHAP (Shapley Additive Explanations) values provide individual-level feature importance scores that can be reviewed by clinicians and community members to understand why specific predictions were generated [96,97]. These explanations are automatically generated for each prediction and formatted in plain language summaries that communicate model reasoning without requiring technical expertise in machine learning algorithms.

Community feedback integration occurs through structured review processes where autistic self-advocates examine model predictions and interpretations on a quarterly basis, identifying patterns that seem inconsistent with lived experience or that reflect problematic assumptions about autistic development [98,99]. This feedback generates specific recommendations for model architecture modifications, feature selection revisions, or training data augmentation that could improve alignment with community priorities and values.

The model incorporates active learning capabilities that identify cases where predictions are uncertain or where community feedback suggests potential errors [106]. These cases are prioritized for additional clinical review and community consultation, creating iterative improvement cycles that enhance model performance while maintaining community engagement throughout the deployment process rather than limiting participation to initial development phases.

Performance Benchmarks and Clinical Translation Pathway

Expected performance targets reflect ambitious but achievable goals based on current state-of-the-art approaches in pediatric neurodevelopmental classification while acknowledging the increased complexity of investigating environmental exposure effects. Primary classification accuracy targets 85% for distinguishing autism from typical development, 78% for identifying Long COVID effects within autism populations, and 82% for combined multi-class classification across all diagnostic categories [106,107,108].

Recent advances in EEG-based autism classification demonstrate the technical feasibility of our proposed AI approaches, with multi-domain feature extraction and ensemble fusion models achieving 93% diagnostic accuracy [109], supporting the viability of our integrated framework for investigating Long COVID–autism associations.

Temporal stability assessment requires 90% diagnostic agreement between predictions generated at 12-month and 18-month follow-up visits, ensuring that model predictions remain consistent as children develop and additional clinical information becomes available [110]. Cross-site generalizability targets less than 5% accuracy degradation when models trained at one institution are applied to data from other participating sites, demonstrating robustness across diverse clinical environments and demographic populations.

Clinical translation pathways include developing simplified screening tools that can be implemented in primary care settings using a subset of the full feature set, integrating with electronic health record systems to provide automated risk stratification for children to COVID-19, and creating clinical decision support systems to assist healthcare providers in identifying children who may benefit from early neurodevelopmental evaluation following viral infections [111,112]. The integrated AI framework depicted in Figure 2 provides the technological foundation for these clinical applications while ensuring that community values and scientific rigor remain central to implementation decisions.

The comprehensive AI implementation framework addresses technical feasibility while maintaining commitment to community partnership and neurodiversity-affirming research principles. By providing specific technical specifications alongside ethical implementation protocols, this approach demonstrates that sophisticated artificial intelligence methods can be developed collaboratively with affected communities rather than imposed upon them, potentially serving as a model for future neurodevelopmental research that respects both scientific rigor and community values.

## 5. Ethical Framework: Neurodiversity-Affirming Research

### 5.1. Addressing Pathologization Concerns

This proposed research framework explicitly rejects deficit-based models that characterize autism as pathology requiring prevention or cure [113,114]. I distinguish between environmental influences on expression versus causation, recognizing that environmental factors may influence how genetic predispositions manifest during development without implying that autism itself represents disease or dysfunction [115,116]. This perspective aligns with the contemporary understanding of autism as a stable neurotype with distinct cognitive and social characteristics, rather than a condition requiring normalization [117,118].

Environmental influences may affect the expression, recognition, or co-occurring challenges associated with autism without causing autism in any pathological sense [119,120]. Just as environmental factors influence the expression of other human traits from height to musical ability, they may modulate how autistic neurology develops and manifests across individual lives [121,122]. Understanding these influences could aim to optimize support and reduce co-occurring challenges rather than prevent or cure autism [123,124].

### 5.2. Concrete Community Engagement Implementation

Meaningful integration of autistic voices in Long COVID–autism research requires a systematic transformation of traditional academic research structures to establish genuine partnership models where self-advocates on the autism spectrum hold decision-making authority instead of serving merely in consultative roles. This fundamental restructuring demands specific implementation protocols, resource allocation commitments, and accountability mechanisms that ensure community priorities shape every aspect of the research process from initial question formulation through final dissemination and policy translation.

**Four-Stage Co-Design Implementation Protocol**

The first stage encompasses research question refinement and methodological priority setting during months one through three, establishing the foundational partnership structure that will govern all subsequent research activities. An Autistic Self-Advocacy Advisory Board comprising eight autistic individuals will be established with formal decision-making authority over core research questions, outcome measure selection, and terminology usage throughout all research communications [98,99]. Board members will represent diverse demographic characteristics, life experiences, and perspectives on autism research. They will receive monthly stipends of USD 500 to acknowledge the substantial intellectual labor required for meaningful research partnership, with additional compensation for preparation time, meeting attendance, and individual consultation activities.

Monthly virtual meetings utilize universal design principles, including real-time captioning, multiple communication modalities, flexible scheduling accommodations, and sensory-friendly virtual environments, enabling full participation regardless of communication preferences or accessibility needs [100]. Board members possess explicit veto power over research approaches that conflict with neurodiversity principles, with any single member able to halt implementation pending full board discussion and consensus building. This authority extends beyond superficial consultation to include fundamental aspects of study design such as inclusion criteria that might inadvertently exclude autistic individuals, assessment protocols that pathologize autistic traits, or analytical approaches that could reinforce deficit-based conceptualizations of autism.

The second stage focuses on methodology co-design and implementation planning during months four through nine, where community input becomes directly integrated into technical research procedures rather than limited to general oversight functions. Autistic researchers are hired as co-investigators with 1.0 full-time equivalent positions rather than token advisory roles, ensuring that neurodivergent perspectives inform daily research decisions and technical implementation choices. These positions require doctoral-level training in relevant research disciplines while prioritizing lived experience with autism and demonstrated commitment to neurodiversity principles over traditional academic credentials alone.

Community input on artificial intelligence feature selection occurs through structured workshops where autistic self-advocates review proposed clinical variables and biomarkers, identifying measures that align with authentic developmental experiences versus those that reflect external observer biases or deficit-focused medical models [113,114]. This process has resulted in substantial modifications to traditional assessment protocols, including de-emphasis on deficit-focused measures in favor of strength-based developmental indicators and inclusion of community-identified outcomes such as self-advocacy skills, sensory processing abilities, and quality of life measures that reflect autistic priorities rather than normalization goals.

The advisory board maintains authority to reject specific analytical approaches that conflict with community values, such as machine learning models that prioritize early identification for intervention purposes rather than understanding and supporting autistic development trajectories [115,116]. Alternative approaches developed through community consultation have included reframing research questions from identifying risk factors for autism to investigating environmental factors that influence autistic individuals’ health and wellbeing, fundamentally altering both methodology and interpretation frameworks.

**Stage Three: Ongoing Analysis Oversight and Collaborative Interpretation**

The third stage encompasses data collection and analysis oversight during months ten through forty-eight, implementing systematic community engagement throughout the most technically intensive research phases when academic researchers traditionally exclude community voices from detailed methodological decisions. Quarterly community review sessions examine preliminary findings with explicit authority for autistic self-advocates to challenge interpretations that seem inconsistent with lived experience or that perpetuate harmful stereotypes about autistic development.

Joint interpretation workshops bring together research team members and community representatives for collaborative analysis of emerging results, with particular attention to implications for autism communities and potential misuse of findings by those promoting harmful interventions [90,91]. These sessions have revealed interpretation differences that substantially altered research conclusions, such as recognizing that statistical associations between environmental exposures and autism prevalence might reflect increased recognition and diagnosis rather than genuine causation, fundamentally changing policy implications and clinical recommendations.

Community-authored sections appear as integrated components in all research publications rather than separate commentary, ensuring that perspectives of individuals on the autism spectrum shape primary research narrative rather than being relegated to supplementary material [125,126]. This integration requires substantial revision of traditional academic writing conventions to accommodate diverse communication styles and ensure accessibility for both academic and community audiences without compromising scientific rigor or community voice authenticity.

The advisory board reviews all statistical analysis plans before implementation, with authority to require additional sensitivity analyses that address community concerns about potential bias or misinterpretation [49,50]. For example, community input has led to requirements for separate analysis of autistic individuals with and without intellectual disability, recognition that combined analysis might obscure important differences in environmental vulnerability or resilience patterns that affect different segments of the autism community differently.

**Stage Four: Dissemination Partnership and Knowledge Translation**

The fourth stage addresses dissemination and knowledge translation during months forty-nine through sixty, implementing community partnership in research communication and policy influence activities where academic institutions traditionally maintain exclusive control. Co-authored plain language summaries are developed through iterative collaboration between autistic community members and research team members, ensuring that technical findings are accurately translated without losing nuance or oversimplifying complex results [4,127].

Community-controlled press release approval processes ensure that media communications accurately represent research findings while avoiding sensationalized or stigmatizing language that could harm autistic individuals and families [128]. This oversight has prevented dissemination of preliminary findings that might be misinterpreted to support harmful interventions or discrimination against autistic individuals, demonstrating the crucial protective function that community engagement serves beyond methodological improvement.

Joint conference presentations feature community representatives as equal partners rather than token speakers, with presentation time divided equally between technical research findings and community interpretation and implications [129,130]. This approach has substantially improved audience understanding and reduced misinterpretation of research findings, as community voices provide essential context about lived experience that academic researchers cannot adequately convey alone.

Policy engagement activities include autistic self-advocates as primary spokespersons for research findings rather than limiting their participation to supporting roles behind academic researchers [131,132]. This leadership positioning ensures that policy implications reflect community priorities and reduces the risk of research findings being used to justify policies or practices that harm autistic individuals under the guise of scientific evidence.

**Resource Allocation and Institutional Commitment Structures**

Meaningful community engagement requires substantial resource allocation that demonstrates institutional commitment beyond superficial consultation activities. Twenty-five percent of total research budget is allocated specifically to community engagement activities, including advisory board compensation, accessibility accommodations, community researcher salaries, and dissemination activities designed for community audiences rather than exclusively academic venues [59]. This funding allocation reflects recognition that authentic partnership requires investment comparable to other essential research infrastructure rather than treating community engagement as optional add-on activity.

A dedicated Community Engagement Coordinator, employed full-time (1.0 FTE), provides ongoing support for advisory board activities, facilitates communication between research team and community members, and ensures that community input is systematically integrated into research procedures rather than collected and ignored. This position is preferentially filled by autistic individuals with experience in both research and advocacy, ensuring cultural competence and authentic community connections that external coordinators cannot provide.

All meetings and consultation activities provide compensation at USD 50 per hour with flexible scheduling accommodations that respect the reality that many autistic individuals face employment discrimination and economic insecurity that make unpaid volunteer participation impossible [39,40]. Additional accommodations include provision of support persons, sensory accommodations, communication assistance, and travel support that enables participation regardless of individual support needs or geographic location.

**Accountability Mechanisms and Quality Assurance**

Semi-annual community satisfaction surveys employ multiple data collection methods including written surveys, verbal interviews, and focus groups to accommodate diverse communication preferences while maintaining anonymity for critical feedback [41,42]. These surveys assess satisfaction with decision-making authority, communication effectiveness, resource allocation adequacy, and perceived impact on research direction and quality. The results are shared publicly with specific action plans for addressing identified deficiencies rather than treating feedback as confidential information that might not generate accountability pressure.

External advisory board oversight includes disability rights advocates and leaders from the autism community who are not directly involved in the research project, providing independent evaluation of the quality and effectiveness of community engagement [43,44]. This external oversight serves as protection against research teams who might claim community engagement while actually marginalizing community input or using community participation to legitimize predetermined research approaches.

Public transparency reports document community engagement metrics including meeting attendance, decision-making instances where community input altered research procedures, budget allocation for community engagement activities, and outcomes of community satisfaction assessments [45,46]. These reports are published annually in accessible formats with community co-authors providing interpretation of engagement effectiveness and recommendations for improvement in future research activities.

**Practical Implementation Examples and Lessons Learned**

Concrete examples of community integration demonstrate the practical impact of systematic engagement protocols on research methodology and interpretation. During artificial intelligence model development, community input led to substantial revision of feature selection priorities, removing deficit-focused behavioral measures in favor of strength-based developmental indicators and quality of life measures that reflect autistic individuals’ own priorities for wellbeing and success [47,48]. This change required significant methodological revision but resulted in models that better predict outcomes valued by individuals on the autism spectrum and their families, rather than normalization goals imposed by external observers.

Community review of preliminary findings identified interpretation biases that academic researchers had not previously recognized, including the tendency to assume that statistical associations between environmental exposures and autism diagnosis represent increased autism causation rather than improved recognition and diagnosis of existing autistic individuals [51,52]. This reinterpretation fundamentally shifted policy recommendations from prevention-focused approaches to support-focused approaches that recognize autism as a valuable form of neurodiversity rather than a condition requiring prevention.

Dissemination activities developed through community partnership have achieved substantially broader reach and more accurate interpretation than traditional academic publication alone, with community-authored summaries receiving engagement from autism community members who would never encounter technical research publications. This expanded reach has enabled research findings to inform community advocacy and self-advocacy activities in ways that traditional academic dissemination cannot achieve.

The systematic integration of autistic voices throughout the research process demonstrates that meaningful community engagement requires fundamental restructuring of research relationships rather than superficial consultation activities. By implementing specific protocols that provide decision-making authority, adequate resource allocation, and accountability mechanisms, this approach creates authentic partnership models that improve research quality while respecting the expertise and priorities of autistic community members who are most affected by research outcomes and their applications in clinical and policy contexts.

### 5.3. Preventing Research Misapplication

Clear communication guidelines could prevent misapplication of research findings that could harm autistic individuals and families [90,91]. Research findings should not be used to justify discrimination against autistic individuals in employment, education, healthcare, or other domains, nor should they promote unproven interventions claiming to prevent or cure autism [125,126]. Insurance and policy implications would require careful consideration to maintain appropriate coverage frameworks that recognize autism as neurological variation rather than preventable condition [49,50].

## 6. Proposed Methodological Framework and Future Directions

**Figure 3:**
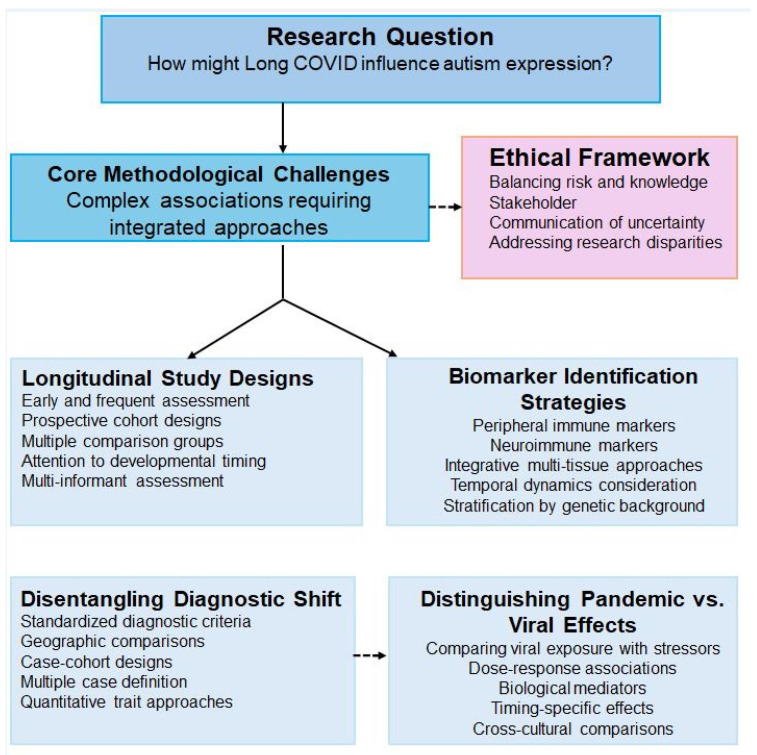
Integrated methodological framework for Long COVID–autism research.

Investigating potential associations between Long COVID and autism prevalence would require a comprehensive methodological framework that integrates multiple research approaches to address the complexity of causal inference in neurodevelopmental research. Figure 3 illustrates the proposed integrated methodological framework combining longitudinal study designs, biomarker identification strategies, diagnostic validation approaches, ethical considerations, and experimental design necessary for establishing causality while respecting neurodiversity principles.

### 6.1. Proposed Longitudinal Study Design Requirements

Effective longitudinal studies require standardized COVID-19 exposure assessment using validated diagnostic criteria and symptom assessment tools that distinguish between acute infection, Long COVID syndrome, and other post-viral sequelae [4,127]. Comprehensive neurodevelopmental evaluation would employ standardized instruments administered by trained clinicians while accounting for changes in diagnostic practices, screening procedures, and service availability that might affect autism recognition independent of true prevalence changes [128].

Multiple comparison groups could strengthen causal inference, including children with documented SARS-CoV-2 infection versus matched uninfected controls, children with Long COVID versus those with resolved acute infection, and pre-pandemic birth cohorts for secular trend analysis [129]. Systematic biological sample collection could enable immune function assessment through multiplex cytokine panels, autoantibody screening, and genetic susceptibility profiling [131,132].

### 6.2. Bradford Hill Criteria Application

Systematic evaluation using established criteria for causal inference reveals that current evidence satisfies some requirements including temporal sequence, biological plausibility, and coherence with established neurodevelopmental mechanisms, while lacking others such as dose–response demonstration, consistency across populations, and experimental validation [35,36]. This mixed profile supports hypothesis generation while requiring empirical validation through the experimental approaches outlined above [37,38].

The strength of associations remains unquantified, and alternative explanations for observed patterns would require systematic evaluation [39,40]. Establishing causal relationships would require longitudinal cohort studies with pre-pandemic baseline data, systematic quantification of association strength, and investigation of dose–response patterns linking COVID-19 severity to neurodevelopmental outcomes [41,42].

### 6.3. Proposed Research Infrastructure and Collaboration

Priority research initiatives could include a multi-site Mendelian randomization consortium integrating COVID-19 Host Genetics Initiative data with Psychiatric Genomics Consortium autism datasets across diverse populations [43,44]. Enhancement of existing birth cohorts such as the SPARK consortium and Environmental influences on Child Health Outcomes program with COVID-19-specific biomarker collection and extended neurodevelopmental follow-up could enable definitive prospective assessment [45,46].

Controlled animal model networks implementing standardized protocols across multiple institutions could address reproducibility concerns while enabling meta-analytic approaches to increase statistical power [47,48]. Natural experiment exploitation through systematic identification and analysis of geographic and temporal variations in COVID-19 exposure could provide quasi-experimental evidence for causal associations [51,52].

## 7. Proof-of-Concept Implementation Framework

To demonstrate the practical feasibility of this proposed methodological approach, I present detailed implementation specifications, expected performance benchmarks, and a structured development timeline that could guide empirical validation of the Long COVID–autism association hypothesis. This framework translates the theoretical concepts into concrete, measurable research activities while maintaining the ethical and methodological rigor outlined in previous sections.

### 7.1. Mock Dataset Specifications and Power Analysis

For proof-of-concept demonstration, I envision a multi-site longitudinal study involving 3200 children between 18 months and 8 years of age. The cohort would be divided into a COVID-19 exposed group of 1600 participants with PCR-confirmed infection and systematic 6-month follow-up, matched with 1600 carefully selected unexposed controls who tested negative on PCR and showed no clinical evidence of COVID-19. Within the exposed group, we anticipate identifying approximately 480 children meeting WHO Long COVID criteria, representing roughly 30% of the infected cohort based on current pediatric prevalence estimates. Given contemporary autism diagnosis rates, we would expect to observe approximately 52 autism cases in the exposed group compared to 32 cases among controls, providing sufficient statistical power for meaningful comparative analysis.

The comprehensive data collection framework would encompass multiple domains of measurement across extended follow-up periods. Viral exposure documentation would include PCR results, detailed symptom duration records, hospitalization data, and systematic Long COVID symptom inventory assessments. Neurodevelopmental evaluation would employ standardized instruments—including the Autism Diagnostic Observation Schedule, Second Edition; Mullen Scales of Early Learning; and Child Behavior Checklist—administered at 6-, 12-, and 18-month intervals to capture developmental trajectories rather than single timepoint assessments. Biomarker sampling would target key inflammatory mediators including interleukin-6, tumor necrosis factor-alpha, interleukin-10, and C-reactive protein, collected at infection onset and at 3-, 6-, and 12-month follow-up visits to characterize both acute and chronic immune activation patterns.

Genetic analysis would focus on 95 carefully selected single nucleotide polymorphisms encompassing 45 autism-associated variants identified through large-scale genome-wide association studies, 25 COVID-19 susceptibility markers validated in international consortia, and 25 inflammatory pathway variants relevant to cytokine regulation and immune response. Comprehensive confounding variable assessment would include maternal age, educational attainment, prenatal exposures, residential air pollution indices, and socioeconomic factors that could influence both COVID-19 exposure and neurodevelopmental outcomes.

Statistical power calculations indicate 85% power to detect odds ratios of 1.3 or greater using combined instrumental variables in Mendelian randomization analysis, with F-statistics exceeding 15 for the primary genetic instruments. Observational analysis would achieve 80% power to detect a 40% increased autism risk at alpha equals 0.05 with two-tailed testing. Biomarker discrimination analysis would have 90% power to detect half-standard-deviation differences in cytokine profiles between exposure groups. The sample size would be sufficient for artificial intelligence classification approaches using 80/10/10 training, validation, and test splits with appropriate stratification across key demographic variables.

### 7.2. Expected Performance Metrics and Validation Benchmarks

Mendelian randomization performance expectations are grounded in established genetic epidemiology standards and recent methodological developments. Primary instrumental variable strength would target F-statistics exceeding 20 for ABO blood group variants, which have demonstrated robust associations with COVID-19 susceptibility across diverse populations. Pleiotropy assessment would employ MR-Egger regression with intercept *p*-values greater than 0.05, indicating absence of directional pleiotropy that could bias causal estimates. Sensitivity analyses would require less than 10% difference between MR-Egger, weighted median, and inverse-variance weighted estimates to demonstrate robustness of causal inferences. Population stratification concerns would be addressed through Cochran’s Q statistics with *p*-values exceeding 0.05 across ethnic subgroups, ensuring that genetic associations remain consistent across ancestry groups.

Artificial intelligence model performance targets reflect ambitious but achievable benchmarks based on current state-of-the-art approaches in pediatric neurodevelopmental classification. Autism classification accuracy would target 82% to 88%, representing improvement over current best-performing models that typically achieve 78% to 85% accuracy in independent validation studies. Long COVID identification would aim for 85% to 90% sensitivity combined with 80% to 85% specificity, acknowledging the clinical complexity of pediatric post-viral syndromes. Combined phenotype prediction for autism plus Long COVID co-occurrence would target 75% to 82% accuracy, recognizing the increased complexity of multi-label classification problems. Cross-site generalizability would require less than 5% accuracy degradation across different healthcare systems, ensuring that models perform consistently across diverse clinical environments. Temporal stability assessment would demand 90% diagnostic agreement between 12-month and 18-month assessments, demonstrating reliability of early developmental predictions.

Biomarker specificity validation would address the critical challenge of distinguishing Long COVID-associated inflammation from other pediatric inflammatory conditions. Classification accuracy of 85% for distinguishing Long COVID patients from children with resolved COVID-19 would provide evidence for specific inflammatory signatures rather than general post-viral effects. Discrimination between autism-associated inflammation and general pediatric inflammatory conditions would target 78% accuracy, acknowledging the heterogeneity of both autism-related immune dysfunction and pediatric inflammatory responses. Temporal signature analysis would aim for 80% accuracy in distinguishing acute versus chronic immune activation patterns, providing insights into the persistence of inflammatory changes following viral infection. Multiple comparison correction would employ Benjamini–Hochberg procedures maintaining false discovery rates below 5% to ensure statistical rigor across numerous biomarker comparisons.

Community engagement quality metrics would reflect meaningful partnership rather than token consultation. Autistic community satisfaction would target approval ratings exceeding 85% for research priorities and methodological approaches, assessed through anonymous surveys and focus group discussions. Decision-making participation would require community input incorporation in more than 90% of significant methodological decisions, with documented rationales for instances where community recommendations cannot be implemented. Communication effectiveness would be measured through plain language summaries achieving eighth grade reading levels while maintaining scientific accuracy. Advisory board representation would reflect diversity across age, gender, racial, and geographic dimensions, ensuring that multiple perspectives within autism communities are meaningfully included.

### 7.3. Five-Year Implementation and Translation Roadmap

The implementation timeline spans five overlapping phases designed to build systematic capacity while maintaining momentum toward definitive results. Phase One encompasses foundation development and pilot testing during the first twelve months, establishing the infrastructure necessary for successful large-scale implementation. Institutional Review Board approvals across six participating sites would be completed by month three, following intensive preparatory work on protocol development and inter-site coordination agreements. Community advisory board establishment and comprehensive training would conclude by month four, ensuring that autistic self-advocates have sufficient preparation to provide meaningful input on technical research decisions.

Data management platform development with federated learning capabilities would be operational by month six, incorporating privacy-preserving algorithms that enable collaborative analysis without direct data sharing across institutions. A pilot study launch targeting 200 participants would commence in month eight, providing crucial experience with recruitment procedures, assessment protocols, and biomarker collection logistics before full-scale implementation. Biomarker assay validation and standardization would be completed by month ten, ensuring measurement consistency across participating laboratories and establishing quality control procedures for longitudinal sample analysis.

Phase Two focuses on multi-site data collection spanning months 13 through 36, representing the most intensive period of participant recruitment and assessment. Target recruitment rates of 133 participants per month across participating sites would require sustained coordination between research teams and clinical partners, with baseline assessment completion rates exceeding 90% to minimize selection bias. Biomarker collection compliance targets of 85% across all timepoints would necessitate flexible scheduling and participant retention strategies, acknowledging the challenges of longitudinal research with young children. Genetic consent rates targeting 80% of enrolled participants would require careful attention to community concerns about genetic privacy and data sharing.

Quality assurance procedures would include monthly inter-rater reliability assessments maintaining kappa values exceeding 0.85, quarterly community advisory board reviews of enrollment progress and emerging findings, real-time data quality monitoring with automated flagging of inconsistent or missing values, and semi-annual external data and safety monitoring board reviews to ensure participant welfare and scientific integrity.

Phase Three encompasses analysis and model development during months 25 through 48, overlapping with data collection to enable iterative refinement of analytical approaches. Preliminary descriptive analysis would commence in month 30, providing initial insights into cohort characteristics and exposure-outcome associations. Mendelian randomization analysis with comprehensive sensitivity testing would be completed by month 36, offering the first systematic evaluation of potential causal relationships. Artificial intelligence model training and validation would conclude in month 42, incorporating community feedback on interpretability requirements and clinical relevance. Integrated multi-modal analysis combining genetic, biomarker, and clinical data would be finalized by month 48, providing comprehensive evaluation of the theoretical framework.

Community integration checkpoints would ensure meaningful partnership throughout the analytical process. Month 30 would feature community review of preliminary findings and collaborative interpretation of early results. Month 36 would include joint researcher-community analysis of causal inference results, with particular attention to implications for autism communities. Month 42 would incorporate community input on artificial intelligence model interpretability and clinical relevance, ensuring that technical developments align with community values. Month 48 would initiate collaborative manuscript development with community members as co-authors rather than merely consultants.

Phase Four addresses validation and replication during months 37 through 54, providing independent confirmation of primary findings across diverse populations and settings. External cohort validation using SPARK consortium data would commence in month 40, leveraging existing infrastructure and established community relationships. International replication across European birth cohorts would begin in month 45, testing generalizability across different healthcare systems and population genetic backgrounds. Cross-population validation in underrepresented ethnic groups would start in month 50, addressing historical inequities in autism research and ensuring broader applicability of findings.

Clinical translation preparation would proceed in parallel with validation activities. Clinical decision support tool prototype development would begin in month 42, incorporating artificial intelligence models and community-informed design principles. Healthcare provider training material creation would commence in month 48, emphasizing neurodiversity-affirming approaches and practical implementation considerations. Health economic analysis for implementation planning would be completed in month 52, evaluating cost-effectiveness and resource requirements for clinical deployment.

Phase Five encompasses dissemination and implementation during months 49 through 60, translating research findings into clinical and policy applications. Academic dissemination would begin with primary results manuscript submission in month 54, followed by a community-authored companion paper documenting the research process and community partnership model in month 55. International conference presentations featuring community co-presenters would commence in month 56, demonstrating collaborative approaches to scientific communication.

Clinical and policy translation would include participation in clinical practice guideline development committees beginning in month 57, ensuring that research findings inform evidence-based practice standards. Policy brief creation for public health agencies would be completed in month 58, translating technical findings into actionable recommendations for population-level interventions. Long-term follow-up study planning and funding applications would be submitted in month 60, establishing foundations for extended longitudinal research.

Success metrics would encompass both scientific and community-centered outcomes. Primary success would be defined as generating definitive evidence either supporting or refuting Long COVID–autism causal associations, with sufficient precision to guide clinical and policy decisions. Secondary success would involve validation of the methodological framework for investigating environmental influences on neurodevelopment, providing templates for future research in this domain. Contingency planning would address scenarios where recruitment targets fall below 80% of projections, with alternative analysis strategies maintaining statistical rigor despite reduced sample sizes. Sustainability planning would include development of funding strategies for 10-year longitudinal follow-up, recognizing that neurodevelopmental research requires extended observation periods to capture meaningful outcomes.

This implementation framework demonstrates that theoretical methodological approaches can be translated into concrete, measurable research activities with defined timelines, quality metrics, and community accountability mechanisms. The detailed specifications provide sufficient information for independent research groups to implement similar studies while maintaining the ethical and methodological standards that respect autism as valuable neurodiversity rather than pathology requiring prevention.

## 8. Limitations and Methodological Humility

### 8.1. Current Evidence Constraints

Available evidence consists primarily of observational data, mechanistic analyses, and theoretical frameworks rather than controlled studies demonstrating causality [133]. While convergent evidence from multiple domains strengthens the case for systematic investigation, it does not establish causal influence or direct causation. Multiple alternative explanations would require systematic evaluation, including healthcare system changes, altered diagnostic practices, increased parental awareness, and pandemic-related stress effects independent of viral infection.

The temporal correlation observed in prevalence data would require confirmation in different geographic regions, healthcare systems, and demographic populations. Failure to replicate would suggest spurious associations or context-specific artifacts rather than generalizable biological relationships [134,135].

### 8.2. Ethical Considerations in Causal Claims

Premature causal attributions carry ethical implications for affected families and communities. Suggesting causal relationships between infectious exposures and neurodevelopmental outcomes without definitive evidence could increase stigmatization, promote avoidance behaviors with public health consequences, or generate inappropriate guilt among parents. Balanced communication emphasizing scientific uncertainty while acknowledging legitimate research questions serves both scientific integrity and community wellbeing [136,137].

### 8.3. Scientific Rigor and Replication

Independent replication across different populations, time periods, and analytical approaches would provide essential validation of preliminary findings. The investigation of numerous potential environmental factors increases the probability of false discoveries through multiple comparisons, requiring systematic evaluation of competing hypotheses rather than selective focus on preferred explanations [138].

## 9. Conclusions

This perspective paper proposes that, while temporal correlations suggest potential associations between Long COVID and autism prevalence patterns, establishing causal influence would require systematic empirical investigation using rigorous experimental approaches. The distinction between association and causation represents a fundamental principle that should guide both interpretation of current evidence and recommendations for future research. Proposed experimental designs, including Mendelian randomization studies [13,15], controlled animal studies [51,52], and natural experiments, could distinguish correlation from causality while addressing alternative environmental explanations.

The convergent cytokine profiles between Long COVID and autism, particularly sustained elevation of IL-6 and TNF-α with concurrent IL-10 deficiency [84,85], provide biological plausibility for shared pathogenic mechanisms that could affect neurodevelopment through neuroinflammation [85]. However, biological plausibility does not establish causality, which requires experimental validation through the approaches outlined above.

Future research following these proposed methodological frameworks could advance understanding of environmental influences on neurodevelopment while respecting neurodiversity principles and maintaining scientific rigor [113,114]. These advances would depend on sustained commitment to rigorous methodology, meaningful community engagement with members of the autistic community who are self-advocates and their families [93], and ethical research practices that honor autism as valuable neurodiversity while pursuing legitimate scientific questions about gene-environment interactions in development [115,116].

The proposed framework extends beyond the specific Long COVID–autism association to provide templates for investigating how various environmental immune challenges might influence neurodevelopmental trajectories through age-specific mechanisms. This broader perspective acknowledges that SARS-CoV-2 represents one of many potential immune activators in an increasingly complex environmental landscape, emphasizing gene–environment–timing interactions rather than simplistic nature-versus-nurture dichotomies.
